# Associations between maternal personality dysfunction and emotion suppression and adolescent emotion suppression

**DOI:** 10.1186/s40479-024-00273-0

**Published:** 2024-11-27

**Authors:** Jennifer J. Phillips, Cynthia L. Smith, Martha Ann Bell

**Affiliations:** 1 Department of Psychology, Virginia Tech, Blacksburg, VA 24060 USA; 2 Department of Human Development and Family Science, Virginia Tech, Blacksburg, VA 24060 USA

**Keywords:** Adolescence, Maternal parenting, Emotion regulation, Personality dysfunction, LPFS, Emotional dysfunction

## Abstract

**Background:**

Adaptive strategies of emotion regulation are important for adolescents, as maladaptive strategies of such can manifest as psychopathology that is sometimes severe. Individual biological characteristics and influences from peers have been shown to have an effect on the development of emotion regulation strategies in adolescents. Maternal factors, however, have received less attention in this age group regarding how they might predict emotion regulation in adolescents. Given that prior work has demonstrated that certain maternal factors, like emotion regulation and personality, play a crucial role in the development of emotion regulation strategies in early childhood, we sought to examine these associations in adolescents in our current study.

**Methods:**

Adolescents and their mothers (*n* = 123) both self-reported data on their own emotion regulation, and mothers also self-reported data on their own personality dysfunction. We operationalized maternal and adolescent emotion regulation as emotion suppression, a maladaptive emotion regulation strategy that is commonly used by adolescents.

**Results:**

Our data demonstrated that both maternal emotion suppression and interpersonal personality dysfunction were positively associated with adolescent emotion suppression. No associations among maternal intrapersonal personality functioning and adolescent emotion suppression were detected.

**Conclusions:**

Maternal personality dysfunction and emotion suppression both independently predicted adolescent emotion suppression use. These results support the idea that maternal characteristics play a role in shaping emotion regulation in adolescence.

## Background

Adolescents who engage in maladaptive emotion regulation strategies are at risk for developing psychopathology that can become severe [35], highlighting the importance of understanding factors that might influence emotion regulation development during adolescence. Research has focused on intrinsic influences of emotion regulation in adolescence, given its developmental trajectory [4]. External variables, such as maternal emotion regulation and personality, however, are equally important to consider when it comes to adolescent emotion regulation [3, 49], but this research is overall limited. Expanding the understanding of the importance of maternal characteristics on adolescent emotion regulation may have implications for intervention and prevention work regarding the psychopathological manifestations that maladaptive emotion regulation strategies can take [13]. Thus, the goal of our current study was to extend this work in adolescent-aged samples.

### Emotion regulation in adolescence

Adolescents regulate their emotions by engaging in any of several strategies that help them to either upregulate or downregulate emotional responses to be appropriate for their current goals [21]. For example, adolescents can use emotion regulation strategies of cognitive reappraisal (CR; reinterpreting an emotional situation to change its meaning and impact) or emotion suppression (ES; stopping or suppressing an emotional response). CR is typically associated with better outcomes, and ES is associated with less optimal outcomes [10, 21]. Although adolescents are capable of using both CR and ES, ES is more commonly used in this age group, as it requires fewer cognitive resources and less brain maturation to use than CR [22, 27]. Additionally, prior research has not identified associations among maladaptive maternal factors (such as what we examined in our current study) and adolescent CR use, but has noted strong associations with adolescent ES use [24]. Therefore, we focused on ES in our current study.

Adolescents who frequently engage in maladaptive emotion regulation strategies, such as ES, are at risk for developing psychopathologic outcomes, including anxiety, aggression, eating pathology, substance use, depression, suicidal ideations, and self-injury behaviors [29, 35, 50]. Problems associated with maladaptive emotion regulation strategies can also persist into adulthood and manifest as personality disorders, like borderline personality disorder (BPD [42],). Given these severe maladaptive outcomes, it is important to understand how ES develops during adolescence and the extrinsic variables that might influence such.

### Maternal factors and adolescent emotion regulation

Emotion regulation begins to develop during infancy within the context of the mother-infant attachment relationship [11]. As children get older, emotion regulation becomes more intrinsic, meaning that they rely less on external sources to help them with emotion regulation [4, 59], and these intrinsic factors are the focus of most of the extant research in this area [49]. Although some evidence has suggested that that adolescents become more independent and individuate themselves from their parents (e.g., [26]), external factors remain highly influential in the continued development of emotion regulation in adolescence.

Indeed, research has demonstrated that the context of the mother-adolescent relationship is important in considering the development of emotional dysregulation and related outcomes (like BPD) in adolescents and emerging adults [14, 32]. Further, mothers may play an important role in emotional socialization in adolescent aged children (e.g., [9]). Despite this, there are some factors that remain under investigated in terms of their empirical associations with adolescent emotion regulation, such as maternal emotion regulation and personality, thus the main goal of the current study. We focused specifically on maternal emotion regulation and personality, as these factors have been widely examined in the early childhood literature (e.g., [20]).

### Maternal emotion regulation

In research spanning early childhood through adolescence, it has been proposed that mothers model emotion regulation behaviors which are imitated by their children [36, 37]. This work suggests that emotion regulation behaviors used by adolescents have roots in what their mothers demonstrated to them during infancy and early childhood. Indeed, in early childhood, there is a direct association between maternal and child emotion regulation behaviors [55] and longitudinal work has shown that preschoolers whose mothers exhibit adaptive emotion regulation strategies also have adaptive emotion regulation strategies when they are in middle childhood [3].

In adolescence, maternal emotion regulation strategies have been established as a reliable predictor of emotion regulation strategies in girls [15]. Other work examining the associations between maternal and adolescent emotion regulation has done so within the context of moderation. For example, mothers with less adaptive emotion regulation strategies tend to have adolescents with similarly less adaptive emotion regulation strategies, but only when mothers are high in hostile and rejecting attitudes towards their adolescents [48]. Despite the extant literature demonstrating that maternal emotion regulation influences adolescent emotion regulation, there is little work overall that focuses on mother-adolescent dyads and emotion regulation, specifically when it comes to evaluating how maternal emotion regulation strategies and personality influence adolescent emotion regulation.

### Maternal personality

Maternal personality plays a role in predicting child emotion regulation strategies, but this work has been primarily focused on personality disorders or negatively valanced temperament traits and examines these associations in early childhood [5, 52]. For example, infants of mothers with BPD tend to have difficulties with emotion regulation, as these mothers tend to inappropriately react to their infants’ emotional responses [28]. Regarding temperament traits, mothers with high levels of temperamental negative affect tend to have infants and young children who cannot regulate their emotions effectively, putting them at risk for developing maladaptive outcomes [33].

The presence or absence of maladaptive levels of negatively valanced traits or a personality disorder does not sufficiently capture maternal personality dysfunction in daily contexts with children [34]. The extant literature is not only limited in the sense that there is a lack of research with adolescent samples, but also in terms of how maternal personality has been operationalized, highlighting the need to use additional measures of personality in maternal samples. We used the Level of Personality Functioning Scale (LPFS [6],) in our current study to assess maternal personality to address these gaps in the literature.

The LPFS assesses personality dysfunction in daily contexts and explains the degree of severity in personality impairment [2]. The LPFS was developed to capture the core of personality dysfunction, defined through intrapersonal (i.e., identity and self-direction) and interpersonal (i.e., empathy and intimacy) impairments as a clinical measure of personality disorders, with higher levels on the measure indicating higher levels of personality dysfunction. Although originally developed as a clinician-report measure of personality disorders, several self-report measures of the LPFS have since been developed [45] and can be used as a valid measure of personality dysfunction in non-clinical samples [44]. Further, work from our group has demonstrated that the LPFS can be validly used in maternal samples. Specifically, we demonstrated that maternal reports on a self-report brief form of the LPFS (LPFS- Brief Form 2.0 [56],) are associated with maternal-reported internalizing and externalizing behaviors in adolescents [40], both of which may be associated with difficulties in emotion regulation [1]. This work was limited, however, as was only focused on maternal report of adolescent behavioral problems and did not actually assess adolescent emotion regulation. We aimed to remedy this in our current study by examining how maternal reports on the LPFS- Brief Form 2.0 relate to adolescent reports of emotion regulation.

Regarding the way that the LPFS is typically used and scored, it has been recommended that the LPFS be represented as a single personality dysfunction score, as opposed to separating it into an intrapersonal and interpersonal score in general and clinical adult populations [7]. Research looking at the use of the LPFS in maternal samples is limited, however; so, there is no certainty as to whether or not this recommendation is appropriate for maternal-specific samples as well. Specifically, regarding the dimensions of the LPFS (i.e., intrapersonal and interpersonal), the *interpersonal* features of empathy and intimacy are important factors in a mother–child relationship (e.g., [30, 58]). Thus, it may be that the interpersonal dimension is a better indicator of personality functioning in maternal samples, as opposed to the intrapersonal dimension or the entire measure. Therefore, in our current study, we opted to assess the LPFS in terms of the individual dimensions (i.e., use one score for intrapersonal functioning and one score for interpersonal functioning).

### The current study

The understanding of the complex associations among maternal characteristics and adolescent ES is underdeveloped. Specifically, the extant work has demonstrated differences in the ways in which maternal ES seem to predict adolescent ES, with some researchers showing direct effects [15] and others interactive effects [48]. Additionally, the work examining how maternal personality relates to adolescent ES focuses on maladaptive levels of negatively valanced personality traits and/or the presence or absence of a disorder [28, 33], which does not provide context for how maternal personality in daily contexts might relate to ES development in adolescents. With our current study, we addressed these limitations by focusing on these associations in a sample of adolescents and their mothers. Further, we examined both direct and interactive effects of both maternal ES and personality, based on prior work demonstrating intricately intertwined associations among these variables (e.g., [5, 52], and utilized a measure of personality designed to assess personality dysfunction in daily intrapersonal and interpersonal contexts. We opted to examine the two LPFS dimensions (i.e., intrapersonal and interpersonal) independently, given prior work suggesting that the factors captured by the interpersonal dimension are more pertinent to the mother–child relationship [30, 58]. We first hypothesized that higher levels of maternal ES would directly predict more adolescent ES, given the positive associations that prior work has demonstrated [15]. Second, we hypothesized that (a) greater maternal intrapersonal and (b) interpersonal personality dysfunction would predict more ES in adolescents, given that prior research has suggested that maladaptive levels of negatively-valanced personality traits and disorders are associated with poorer ER strategies across childhood in younger samples [28]. Finally, after assessing maternal ES and personality dysfunction as independent predictors of adolescent ES, we considered the direct and interactive associations of these variables together in light of the Determinants of Parenting Process Model [5, 52] and examined maternal ES as a moderator in the association between maternal personality dysfunction and adolescent ES. Specifically, our third hypothesis was that higher levels of maternal ES would moderate the associations between (a) more maternal intrapersonal personality dysfunction and adolescent ES, and (b) more maternal interpersonal personality dysfunction and more adolescent ES.

## Method

### Participants

Participants for this study included adolescents and their biological mothers who were part of the Cognition, Affect, and Psychophysiology (CAP) Study, which was the same sample used in our previously published paper examining the effects of maternal LPFS on maternal reported adolescent behavioral problems [40]. Three cohorts of typically-developing children (e.g., normal gestation length, no genetic or chromosomal anomalies, no developmental diagnoses by age 5-months) and their mothers were recruited when the children were infants using flyers, word of mouth, and mailing lists. Participants in the current study make up two of these cohorts, represent half of the original sample, and were recruited from and participated at the same university research laboratory in a college town in the mid-Atlantic region of the United States. The third cohort was recruited by a university research laboratory located in a city in a different mid-Atlantic state and ended the longitudinal study when the children were in middle childhood. The adolescent portion of the CAP Study was approved by the Biomedical Research Alliance of New York Institutional Review Board (protocol #19–030-568/19–352).

Our sample included 123 adolescents and their mothers. Adolescent age ranged from 11 – 18 years (*M* = 14.64 years, *SD* = 1.94 years). Gender was almost evenly split (60 boys, 63 girls), and the majority of the adolescents were White (92.7%) with smaller proportions being identified as Multi-Racial (5.7%) or Asian (1.6). The majority of the sample was non-Hispanic (95.0%). The wide age range of the adolescents was due to several reasons. First, one cohort of children was, on average, 2–4 years older than children in the other cohort due to research funding schedules. Second, one cohort was recruited as infants across an 18-month period, whereas the other cohort was recruited as infants across a 12-month period. Finally, adolescent data collection occurred across a 13-month period of time.

Adolescents in cohort 1 (*n* = 55) and 2 (*n* = 68) did not differ on demographics or on any of the study variables (*p*s > 0.292). Mothers ranged in age from 31 – 59 years (*M* = 45.18 years, *SD* = 1.93 years) at the time of data collection. The majority of mothers identified as White (95.2%) and smaller proportions identified as Asian (2.4%) or Multi-Racial (2.4%). The majority of mothers identified as non-Hispanic (95.2%). In terms of education at the time of data collection, 3.3% of mothers had not completed high school, 12.2% had a 2-year college degree, 32.5% had a 4-year college degree, and 51.2% had a graduate degree (0.8% of mothers did not report their education). Of the 123 adolescent-mother dyads, 79 participated prior to the COVID-19 pandemic between August 2019 and March 2020 either via an in-person lab visit (*n* = 78) or questionnaires only (*n* = 1). The remaining 44 dyads participated between July 2020 and September 2020 via online questionnaires only, as the pandemic required a pause in our in-person data collection.

### Procedure

Adolescents who visited the lab with their mothers prior to the pandemic participated in a protocol lasting approximately three hours, during which adolescents reported on their own ES and mothers reported on their own ES and personality functioning. Additional questionnaires were completed by both mothers and adolescents, and adolescents participated in a battery of cognitive and affective tasks during physiological data collection, which were not included measures in the current study. Dyads who were questionnaire-only families in the study completed either paper form questionnaires that were mailed (*n* = 1; pre-pandemic) or online questionnaires via Qualtrics (*n* = 44; during pandemic). All adolescents and mothers were compensated monetarily for their participation.

### Measures

#### Maternal and adolescent ES

Mothers and adolescents both self-reported their own ES using the Emotion Regulation Questionnaire (ERQ; [21]) The ERQ consists of 10 items that make up a CR (6 items) and ES subscale (4 items). Higher scores on each subscale indicate greater use of that emotion regulation strategy. We only used the ES subscale in our current study. In our sample, the ES subscale demonstrated acceptable internal consistency in both mothers (α = 0.73) and adolescents (α = 0.75). To our knowledge, this original version of the ERQ has not been frequently used in young-adolescent samples, although there is a revised ERQ validated for use in children and adolescents (see [23]). We argue, however, that the original version of the ERQ that we used to assess adolescent ES in our current study was a reliable measure of such, given the similarities between this version and the revised, as well as the moderate-high alpha we observed in our adolescent sample. See Table 1 for descriptive statistics for mother and adolescent ES responses on the ERQ.

**Table 1:** Descriptive statistics and correlations among study variables (After Winsorizing)

	1	2	3	4	5	6	7
1. Adolescent Gender	1						
2. Adolescent Age	-.11	1					
3. Mom Age	-.08	**.40**^b^	1				
4. Adolescent ES	-.04	.10	.06	1			
5. Mom ES	.03	.13	.10	**.20**^a^	1		
6. Mom Interpersonal	-.06	.05	-.02	**.22**^a^	.12	1	
7. Mom Intrapersonal	-.05	> .01	-.10	.04	.17	**.57**^b^	1
Mean	0.51	14.64	45.24	3.79	2.90	8.65	10.55
SD	0.51	1.94	4.92	1.22	1.09	2.69	3.04
Min	11.82	11.82	31.00	1.00	1.00	6.00	6.00
Max	1.00	18.13	59.00	6.75	6.00	16.00	18.00

**Boldface** text indicates significance

*ES *emotion suppression, *Interpersonal* LPFS interpersonal subscale, *Intrapersonal* LPFS intrapersonal subscale. Adolescent sex was reported by mothers with 0 = boy and 1 = girl

^a^indicates significance at the 5% level

^b^indicates significance at the 1% level.

#### Maternal personality dysfunction

Mothers self-reported their personality dysfunction using the Level of Personality Functioning Scale – Brief Form 2.0 (LPFS- BF 2.0; [56]). The LPFS- BF 2.0 is a 12-item self-report measure based on the earlier clinical version of the LPFS that was proposed in Alternative Model of Personality Disorders in the fifth edition of the Diagnostic and Statistical Manual of Mental Disorders (DSM-5,AMPD, [2]). Briefly, the AMPD consists of two criteria- Criterion A, which is the LPFS, and Criterion B, which is a list of pathological personality traits. In our current study, we are focused on Criterion A. Self-report measures of the LPFS are comparable to the original clinician report measure in terms of reliability and validity [45].

The LPFS- BF 2.0 consists of two six-item dimensions capturing intrapersonal (identity, self-direction) and interpersonal (empathy, intimacy) features. The 12-item LPFS- BF 2.0 is scored on a 4-point Likert scale with 1 indicating low to no levels of personality dysfunction (i.e., less dysfunction) and 4 indicating high, disordered levels of personality dysfunction (i.e., more dysfunction). We created two separate scores for mothers—one intrapersonal score and one interpersonal score—by summing items 1–6 and 7–12, respectively. Although prior work has suggested that measures of personality dysfunction, like the LPFS—BF 2.0 are best represented through a single dimension [7], we opted to examine the two dimensions separately in an attempt to understand how different aspects of Criterion A relate to ES, as ours is the first study to our knowledge to assess this. Because of the way that the LPFS- BF 2.0 is scored, it is possible to achieve a total score between 6 (i.e., responding “1” to each item) and 24 (i.e., responding “4” to each item) for each dimension. Some research has suggested that scoring between a 2 and a 3 on the majority of the LPFS- BF 2.0 items (for a total score between 12 – 18 on each individual dimension) is indicative of personality pathology [56]. Our sample had acceptable internal consistency for both the intrapersonal dimension (α = 0.77) and the interpersonal dimension (α = 0.78). For simplicity, we refer to the LPFS- BF 2.0 as the LPFS, and descriptive statistics for this measure are shown in Table 1.

### Data analysis plan

#### Preliminary analyses

All analyses were conducted using SPSS version 29 [25]. Prior to analyses, the data were inspected for outliers and bivariate correlations among the study variables and demographic variables (i.e., adolescence gender, adolescent age, maternal age) were examined. Outliers were defined as values that fell ± 3 standard deviations of the mean, and one variable (maternal interpersonal personality functioning) met this criterion. Because of this, we winsorized that variable, which is an outlier management technique that replaces outliers with the next closest score [47]. We replaced two maternal interpersonal personality functioning scores that were + 3 standard deviations from the mean. The results of the bivariate correlations did not yield any significant associations between demographic factors as control variables to our analyses. The descriptive statistics for the study variables (after winsorizing) and correlations are shown in Table 1. We also assessed for differences between maternal and adolescent reports of ES using a paired samples t-test. There was a significant difference between maternal and adolescent ES use, *t*(122) = -6.71, *p* < 0.001. Specifically, on average, mothers reported less ES use (*M* = 2.90, *SD* = 1.09) than adolescents (*M* = 3.79, *SD* = 1.22).

Because our data collection occurred both before and during the COVID-19 pandemic, we conducted independent samples t-tests to assess potential differences between the study variables based on the pre- or during-COVID group classifications, given the socioemotional and economic stress that the quarantine period of the pandemic had caused for some [41]. No differences in adolescent ES or maternal personality variables were detected (*p* > 0.410). There were, however, differences in maternal ES based on timing of participation. Specifically, mothers who participated *during* the pandemic (*M* = 3.19, *SD* = 1.18), compared to mothers who participated *prior* to the pandemic (*M* = 2.74, *SD* = 1.11), reported higher levels of ES, *t*(121) = -2.25, *p* = 0.026). Because of these differences in maternal ER between our pre- and during-COVID groups, we controlled for COVID group classification (0 = prior, 1 = during) in our analyses.

Because prior work has demonstrated that maternal ES plays a more prominent role in girls than boys (e.g., [15]), we conducted the analyses once controlling for adolescent gender and once without. The analyses that did control for child gender did not demonstrate gender to play a role in ES (all *p*s > 0.22) and the results did not change with gender controlled for. Thus, we reported only the results without controlling for gender for the sake of conciseness.

#### Regression analyses

To test our hypotheses, we used a hierarchical linear regression model with adolescent ES as the dependent variable. In Step 1, we included COVID group classification and in Step 2, we included maternal ES, intrapersonal personality dysfunction, and intrapersonal personality dysfunction. In Step 3, we included interactions between maternal ES and intrapersonal personality dysfunction and between maternal ES and interpersonal personality dysfunction to examine moderation, testing Hypothesis 3. The Type I error rate was set to 5% for probing significant effects. We also examined the regression model for multicollinearity by evaluating the variance inflation factor (VIF), the condition index (CX) and the variance proportion (VP) for each variable in each step of the model. Multicollinearity was defined according to established guidelines- VIF ≥ 10, CX ≥ 50, VP = 50%—100% [53]. Meeting only one of these conditions was indicative of minor multicollinearity, whereas meeting two or three of these conditions indicated severe multicollinearity [53].

#### Missing data

Of the 123 dyads in our study, two were missing both adolescent and mother ES data (neither dyad returned the questionnaire) and 17 mothers did not complete the LPFS, as it was not added to the study until after data collection for the adolescent portion of the study had a begun. In sum, 104 dyads had complete study data and 19 were missing at least one variable. Because we used multiple regression for our main analyses, we opted to impute missing data points using the expectation–maximization (EM) algorithm in SPSS, as listwise and pairwise deletion can bias estimates and limit power [17, 57]. To use the EM algorithm, data must meet the missing completely at random assumption, which ours did, χ^2^(14) = 8.17, *p* = 0.881.

## Results

### Hypotheses 1 and 2: direct effects

Our first hypothesis was that maternal ES would positively predict adolescent ES, and our second hypothesis was that more maternal intrapersonal and interpersonal personality dysfunction would predict more adolescent ES. These results are shown in Table 2, Step 2. Together, maternal ES and both dimensions of the LPFS explained 9% of variance in adolescent ES. Hypothesis 1 was completely supported, as maternal ES did predict more adolescent ES. Hypothesis 2 was only partially supported. Specifically, maternal *interpersonal* personality dysfunction did predict more adolescent ES; however, maternal *intrapersonal* personality dysfunction was not associated with adolescent ES. No evidence of multicollinearity was detected in this model.

**Table 2:** A priori regression model demonstrating independent and interactive maternal predictors of adolescent ES

Step	Variables	β	*p*	95% CI	VIF	CX	VP
1	COVID-19 *F*(1,121) = 0.04, *R*^*2*^ = .000, *p* = .852	0.02	.852	[-0.41, 0.50]	1.00	5.84	0.97
2	COVID-19 Mom ES** Mom LPFS Interpersonal** Mom LPFS Intrapersonal ***F*(2,118) = 3.19, Δ*R***^***2***^ = .089, *p* = .045**	> 0.01 **0.19** **0.27** -0.14	.990 **.040** **.013** .197	[-0.46, 0.45] **[0.10, 0.41]** **[0.26, 0.21]** [-0.14, 0.03]	1.07 1.07 1.57 1.53	1.09 1.43 2.17 6.05	0.97 0.05 0.03 0.00
3	COVID-19 Mom ES Mom LPFS Interpersonal Mom LPFS Intrapersonal Mom LPFS Interpersonal x Mom ES Mom LPFS Intrapersonal x Mom ES *F*(2,116) = 0.06, Δ*R*^*2*^ = .001, *p* = .938	> -0.01 0.19 0.28 -0.14 -0.26 -0.01	.950 .043 .013 .197 .808 .917	[-0.48, 0.45] [0.01, 0.41] [0.03, 0.22] [-0.14, 0.03] [-0.90, 0.07] [-0.07, 0.07]	1.09 1.08 1.64 1.55 1.41 1.34	1.08 1.21 1.44 1.95 2.34 6.23	0.97 0.05 0.02 0.00 0.02 0.00

**Boldface** text indicates significance

*CI *confidence interval, *VIF *variance inflation factor, *CX *condition index, *VP *variance proportions, *ES *emotion suppression, *LPFS *Levels of Personality Functioning Scale

**indicates significance at the 5% level

### Hypothesis 3: interactive effects

Our third hypothesis was that more maternal ES would moderate the associations between (a) more maternal *intrapersonal* personality dysfunction and more adolescent ES and (b) more maternal *interpersonal* personality dysfunction and more adolescent ES. These results are shown in Table 2, step 3. This hypothesis was not supported, as our data did not demonstrate any interaction effects between maternal ES and either personality functioning dimension in the association with adolescent ES.

### Post-Hoc analyses

Although our hypotheses were partially supported, there are potential concerns regarding low statistical power for the testing of our models. Additionally, we wanted to separate the dimensions of the LPFS due to our predictions regarding the relevance of the interpersonal dimension in mother-specific populations, but we acknowledge that this could raise questions, given that prior work has recommended using the LPFS as a holistic measure [7]. To account for these possible concerns, we conducted a series of post-hoc analyses. Specifically, we conducted two power analyses (i.e., one post-hoc power analysis for the previously tested models and one a priori power analysis for the post-hoc models). In addition, we and ran three additional regression models (i.e., one with intrapersonal dysfunction, one with interpersonal dysfunction, and one with the whole LPFS) to ensure that our results from our a priori models were not influenced unduly by our decision to split the dimensions of the LPFS.

### Power analyses

 For our post hoc power analysis, we used R for Macintosh [43] to calculate the achieved power based on our effect size (i.e., Cohen’s *f*^2^). This power analysis was based on step 3 of our hierarchical regression model (Table 2), and thus included six predictors, two moderators, a sample size of 123, and an observed *R*^2^ of 0.099. The effect size was calculated to be *f*^2^ = 0.110. At the 5% level, the analysis revealed that the achieved power was 0.954, indicating that there was a 95.4% probability of detecting this effect at the sample size of 123. Although the probability was high, the size of the detected effects of maternal personality dysfunction and ES are low-moderate [12], indicating, at best, a moderate impact on predicting adolescent ES. Thus, we proceeded with our post-hoc plan to run the additional regression models mentioned above that buy back some degrees of freedom by including fewer predictors in the model.

For our a priori power analysis to determine if we had the appropriate sample size to test our three additional regression models, we also used R for Macintosh [43] to calculate the sample size necessary for a 95% probability of detecting a moderate effect (i.e., Cohen’s *f*^2^ ≥ 0.15, Est. *R*^*2*^ = 0.13). These power analyses were based on what would be the third step of our post-hoc models and thus included 4 predictors and one moderator. At the 5% level, the analyses revealed that the sample size necessary for *f*^2^ = 0.15 was *n* = 89. Thus, our sample was appropriately powered for the post-hoc regression models.

### Regression models

We tested three additional regression models with adolescent ES as the dependent variable. Step 1 of each model still included COVID group classification as a covariate. Step 2 of each model included maternal ES and either maternal intrapersonal personality dysfunction (Post Hoc Model 1), maternal interpersonal personality dysfunction (Post Hoc Model 2), or maternal composite LPFS scores (i.e., both intrapersonal and interpersonal dysfunction combined; Post Hoc Model 3) as independent predictors of adolescent ES. Step 3 of each model included one interaction term between maternal ES and the model’s respective personality dysfunction variable.

**Post Hoc Model 1.** The results for Post Hoc Model 1 are shown in Table 3. None of the steps of this model were significant.

**Table 3:** Post Hoc Model 1 demonstrating effects of maternal es and intrapersonal dysfunction on adolescent ES

Step	Variables	β	*p*	95% CI	VIF	CX	VP
1	COVID-19 *F*(1,121) = 0.04, *R*^*2*^ = .000, *p* = .848	0.02	.848	[-0.41, 0.50]	1.00	5.84	.97
2	COVID-19 Mom ES Mom LPFS Intrapersonal *F*(2,119) = 2.71, Δ*R*^*2*^ = .044, *p* = .071	-0.02 0.21 0.04	.807 .028 .691	[-0.52, 0.40] [0.03, 0.43] [-0.06, 0.09]	1.04 1.06 1.01	1.70 5.54 7.67	.52 .68 .01
3	COVID-19 Mom ES Mom LPFS Intrapersonal Mom LPFS Intrapersonal x Mom ES *F*(1,118) = 0.01, Δ*R*^*2*^ = .000, *p* = .916	-0.02 0.21 0.04 0.10	.817 .029 .694 .916	[-0.52, 0.41] [0.02, 0.43] [-0.06, 0.09] [-0.06, 0.06]	1.05 1.06 1.02 1.01	1.67 1.73 5.58 7.70	.48 .29 .00 .00

*CI *confidence interval, *VIF *variance inflation factor, *CX *condition index, *VP *variance proportions, *A *adolescent data, *M *maternal data, *ES *emotion suppression, *Inter *LPFS interpersonal subscale, *Intra *LPFS intrapersonal subscale

**Post Hoc Model 2.** The results for Post Hoc Model 2 are shown in Table 4. Step 2 of this model was significant and a positive low-moderate main effect (Cohen’s *f*^2^ = 0.11) of maternal interpersonal personality dysfunction on adolescent emotion suppression was detected. Unlike our a priori model, however, maternal ES was not associated with adolescent ES.

**Table 4:** Post Hoc Model 2 Demonstrating effects of maternal es and interpersonal dysfunction on adolescent ES

Step	Variables	β	*p*	95% CI	VIF	CX	VP
1	COVID-19 *F*(1,121) = 0.04, *R*^*2*^ = .000, *p* = .848	0.02	.848	[-0.41, 0.50]	1.00	5.84	.97
2	COVID-19 Mom ES Mom LPFS Interpersonal** ***F*(2,119) = 5.72, Δ*R***^***2***^ = .088, *p* = .004**	0.01 0.17 **0.22**	.917 .057 **.016**	[-0.43, 0.48] [-0.01, 0.39] **[0.02, 0.18]**	1.04 1.06 1.01	1.70 5.54 7.67	.52 .68 .01
3	COVID-19 Mom ES Mom LPFS Interpersonal Mom LPFS Interpersonal x Mom ES *F*(1,118) = 0.01, Δ*R*^*2*^ = .000, *p* = .958	0.01 0.17 0.22 -0.01	.925 .059 .018 .958	[-0.44, 0.48] [-0.01, 0.39] [0.02, 0.18] [-0.07, 0.07]	1.05 1.06 1.02 1.01	1.67 1.73 5.58 7.70	.48 .29 .00 .00

**Boldface** text indicates significance

*CI *confidence interval, *VIF *variance inflation factor, *CX *condition index, *VP *variance proportions, *ES *emotion suppression

**significant at the 5% level

**Post Hoc Model 3.** Finally, the results for Post Hoc Model 3 are shown in Table 5. Step 2 of this model was also significant. In this model, however, there was a positive main effect of maternal ES on adolescent ES, but no effect between the maternal LPFS composite and adolescent ES was detected.

**Table 5:** Post Hoc Model 3 Demonstrating Effects of Maternal ES and Composite LPFS on Adolescent ES

Step	Variables	β	*p*	95% CI	VIF	CX	VP
1	COVID-19 *F*(1,121) = 0.04, *R*^*2*^ = .000, *p* = .848	0.02	.848	[-0.41, 0.50]	1.00	5.84	.97
2	COVID-19 Mom ES** Mom LPFS Total ***F*(2,119) = 3.78, Δ*R***^***2***^ = .060, *p* = .026**	-0.01 **0.19** 0.13	.915 **.042** .139	[-0.48, 0.43] **[0.10, 0.41]** [-0.01, 0.08]	1.05 1.07 1.03	1.68 5.60 7.66	.52 .69 .03
3	COVID-19 Mom ES Mom LPFS Total Mom LPFS Total x Mom ES *F*(1, 118) = 0.24, Δ*R*^*2*^ = .000, *p* = .878	-0.01 0.19 0.13 -0.01	.934 .043 .145 .878	[-0.48, 0.45] [0.01, 0.41] [-0.01, 0.08] [-0.04, 0.04]	1.08 1.07 1.04 1.03	1.63 1.78 5.64 7.75	.49 .28 .00 .02

**Boldface** text indicates significance

*CI *confidence interval, *VIF *variance inflation factor, *CX *condition index, *VP *variance proportions, *ES *emotion suppression

**significant at the 5% level

## Discussion

The aim of our study was to address the gaps in the previous literature by assessing how maternal ES and intrapersonal and interpersonal personality dysfunction directly affect adolescent ES. Further, as our current study is the first (to our knowledge) to examine the LPFS in relation to adolescent ES, we also examined whether maternal ES might moderate the association between maternal report on the two LPFS dimensions. Our first hypothesis that maternal ES would directly predict adolescent ES was *fully* supported, our second hypothesis that maternal personality dysfunction would directly predict adolescent ES was *partially* supported, and our third hypothesis that maternal ES would moderate the associations between maternal intrapersonal personality dysfunction and adolescent ES and between maternal interpersonal personality dysfunction and adolescent ES was *not* supported. Even after accounting for effect size and statistical power in our post hoc analyses, we were still only able to demonstrate maternal interpersonal personality dysfunction to be directly associated with adolescent ES. Our results are further discussed below in light of the findings from both our a priori and post hoc models.

### Direct associations

#### Maternal and adolescent ES

Emotion regulation strategies like ES have at least some genetic underpinnings [8, 16]. Because of this, as well as research indicating that preschoolers and school-aged children imitate emotion regulation behaviors from their mothers (e.g., [37], our result that maternal ES positively predicted adolescent ES was not surprising. We were, however, intrigued to see this effect, given that adolescents become more independent and individuate themselves from their parents (e.g., [26]). Nevertheless, we had anticipated this result, as previous research has suggested that maternal emotion regulation is associated with adolescent emotion regulation, particularly in girls [15] and in conjunction with parenting behaviors [48]. The direct associations between maternal and adolescent emotion regulation strategies, however, have not been widely studied in previous research, which is likely due to the notion that emotion regulation becomes a more intrinsic process as children age into adolescence [4, 59]. The positive association between maternal and adolescent ES that we demonstrated in this current study provides evidence that mothers continue to play a role in the development and use of emotion regulation strategies in adolescents.

It must be mentioned, however, that the significant effect of maternal ES on adolescent ES was not consistently detected when we separated the maternal personality dysfunction variables in our post hoc analyses. Specifically, the effect was lost in Post Hoc Model 1, a positive association between maternal ES and adolescent ES was trending (*p* = 0.057) in Post Hoc Model 2, and a positive association between maternal ES and adolescent ES was apparent in Post Hoc Model 3 (*p* = 0.042). Because of these inconsistencies, in addition to the small effect size that was detected in our a priori analyses, we are cautious about interpreting maternal ES to have a significant, positive effect on adolescent ES. More research is certainty warranted to fully understand this association.

#### Maternal personality dysfunction and adolescent ES

We hypothesized that maternal intrapersonal and interpersonal personality dysfunction would both positively predict adolescent ES, as prior research with younger children has suggested that personality disorders or maladaptive levels of negatively valanced personality traits in mothers can be detrimental for effective emotion regulation strategies in children (e.g., [28, 33]. To our best knowledge, there are no studies that examine the role of maternal personality on emotion regulation in adolescent samples. Further, research has also indicated that the presence or absence of negative personality traits or disorders is not indicative of personality dysfunction in daily contexts and relationships [34]. Given these gaps in the extant work, we aimed to not only examine the associations between maternal personality and adolescent ES, but we also utilized the LPFS to examine personality functioning, as opposed to traits and disorders, in order to gain a more holistic view of how maternal personality relates to adolescent ES. In doing so, we hypothesized that maternal dysfunction in both intrapersonal and interpersonal domains would predict higher levels of adolescent ES.

Our results did not demonstrate maternal intrapersonal dysfunction was associated with adolescent ES. Our data did show, however, that maternal interpersonal personality dysfunction was positively associated with adolescent ES. Although we had hypothesized that both dimensions of personality dysfunction would be predictive of adolescent ES, it makes conceptual sense that only interpersonal dysfunction had a role in adolescent ES. Specifically, individuals who have interpersonal personality dysfunction have difficulty forming lasting and meaningful relationships with others [6, 56]. This result suggests that adolescent ES use may be a result in having a mother who lacks in her ability to create a meaningful relationship with her adolescent. Further, because younger children learn emotion regulation strategies by imitating their parents [37], interpersonal personality dysfunction could be associated with maternal ES use, as personality has been related to emotion regulation difficulties in the adult literature [38, 51].

Contrary to our hypothesis, maternal intrapersonal personality dysfunction was not associated with adolescent ES. Intrapersonal personality dysfunction is associated with higher levels of self-dysfunction and psychological distress [6]. Thus, it is possible that maternal intrapersonal personality dysfunction did not predict adolescent ES because it is not concerned with social relationships. In other words, because intrapersonal personality dysfunction primarily affects the relationship with one’s own self, it could be that maternal intrapersonal dysfunction does not influence adolescent ES in the same way that maternal interpersonal dysfunction does.

In our post hoc analyses, we were unable to demonstrate an association between maternal intrapersonal personality dysfunction and adolescent ES (Post Hoc Model 1), nor between the maternal LPFS composite and adolescent ES (Post Hoc Model 3). The positive association between maternal interpersonal personality dysfunction and adolescent ES did, however, remain in Post Hoc Model 2. This result, along with the lack of multicollinearity in our a priori model, suggests that there is something distinctive about the way maternal interpersonal personality dysfunction relates to adolescent ES. Because the underlying traits that make up interpersonal personality dysfunction (i.e., intimacy and empathy; [6]) are traits that are also important for effective parenting and the formation of meaningful relationships between mothers and children (e.g., [31]), we propose that the positive association between maternal interpersonal dysfunction and adolescent ES could be tapping into underlying behaviors necessary for meaningful and effective parenting.

Additionally, it is possible that maternal interpersonal dysfunction is more important when it comes to emotion-regulation based outcomes, such as ES strategies, in adolescents. Indeed, research with maternal parenting and adolescent emotion regulation has demonstrated that impaired mother-adolescent relationships are predictive of maladaptive emotion regulation strategies in adolescents (e.g.,). On the other hand, maternal intrapersonal dysfunction or both maternal intrapersonal functioning and/or composite LPFS scores are more important for predicting other outcomes, such as psychopathology and behavioral problems, as we have demonstrated in our prior work with this same sample (e.g., [40]).

Overall, there may be something unique to maternal interpersonal personality dysfunction and adolescent ES, or at the very least, adolescent emotion-related outcomes. This suggests that perhaps splitting the LPFS (or at least the LPFS- BF 2.0) into two dimensions for assessing the association between maternal personality dysfunction and adolescent outcomes might be more appropriate than using it as a holistic measure. Again, we stress that due to the concerns with power in our a priori analyses, this direct effect should be interpreted with caution. Additionally, because our current study is the first to our knowledge that has demonstrated these results regarding maternal interpersonal personality functioning and adolescent ES, more work is crucial to fully understand the workings of this association.

### Interactive effects

Drawing from the Determinants of Parenting Process Model [5, 52] as a guide, our third hypothesis was that maternal ES would moderate the associations between (a) maternal intrapersonal personality dysfunction and adolescent ES and (b) maternal interpersonal personality dysfunction and adolescent ES. We tested this hypothesis by including interaction terms between both maternal personality dysfunction dimensions and maternal ES in a third step of our regression model. None of the interaction terms in the a priori or the post hoc models, however, were significant. This lack of findings suggests that maternal ES does not moderate these associations.

Although we had demonstrated maternal ES and interpersonal personality dysfunction to be independent, direct predictors of adolescent ES, we included the moderation hypothesis in an attempt to better understand how maternal personality dysfunction might influence adolescent ES, given that this is the first study to our knowledge to examine the associations among maternal personality dysfunction and emotion regulation in any age group. The null result is still an important contribution to the literature, as it suggests that maternal ES and personality dysfunction (specifically interpersonal dysfunction) are independently related to the development of adolescent emotion regulation. More research, however, is needed to fully understand these complex relations in not only adolescents, but also younger children.

### Strengths, limitations, and future directions

Our study had several strengths that make it an important contribution to the existing literature on the effects of maternal ES and personality on adolescent ES. First, adolescents self-reported their own ES, which helped to eliminate bias that might result from parent reports of child ER. One of the major limitations of our prior work examining maternal LPFS and adolescent outcomes was that mothers reported on adolescent outcomes, which means that maternal *perceptions* of adolescent outcomes were examined, which could have swayed our results [40]. Second, the absence of multicollinearity in our model suggested that it may be appropriate to examine the LPFS as separate dimensions in maternal samples. Third, our study adds to the limited literature of the effects of maternal ES and personality on adolescent ES, as prior work has focused on early childhood and infant populations (e.g., [20]). Fourth, we further add to the literature by providing evidence for the importance of considering maternal personality functioning on child outcomes by not relying on trait- and disorder-based operationalizations of personality, which are not holistic assessments of such [34]. Fifth, by using the LPFS as our measure of personality dysfunction, we were able to capture personality in a holistic manner that is representative of personality in daily contexts and relationships [6]. Sixth, we did not demonstrate any gender differences in our current study. Prior research has suggested that maternal ES only predicts adolescent ES in daughters, but not sons [15], so our result adds to the literature by showing that maternal personality and ES play a role in adolescent ES, regardless of gender.

Despite its strengths, our study is not without limitations. First, our study lacked overall racial and ethnic diversity. Although the racial demographics of our participants were representative of the geographic location from which they were recruited, our results may not generalize to more diverse populations. Second, mothers reported relatively low levels of personality dysfunction, and both mothers and adolescents reported relatively low levels of ES, indicating that our sample was overall well adjusted. It is unclear if these results would hold in a sample that may meet clinical levels of personality dysfunction and/or higher levels of ES. Third, our study had lower than desired power, so our results should be interpreted with caution. Finally, we did not examine paternal personality dysfunction or ES in our current study. It has been suggested that fathers in heterosexual parent couples also play a role in emotion regulation development in adolescents, specifically by serving as models of activation of emotion regulation [54]. Additionally, fathers in gay parent couples may serve as adaptive examples of coping and autonomy for children and adolescents, leading to stronger emotion regulation skills in middle childhood, compared to heterosexual parent family structures [19]. This work suggests that there are indeed important paternal features that influence emotion regulation development in children and adolescents, so by focusing only on mothers in the current study, we were not able to address an entire subset of research.

Future work should aim to remedy the above limitations by recruiting a larger and more diverse sample, recruiting clinical samples, and including father reports of personality dysfunction and ES in addition to mothers. Other work might consider collecting LPFS data from both adolescents and their parents in order to relate individual personality to the development of ES. As mentioned, personality is a predictor of emotion regulation strategies in both children and adults [27]. Personality also has genetic underpinnings [46], and some work has demonstrated that undergraduate-aged individuals rate their own personality (using the LPFS) similarly to that of their parents [44]. Adding adolescent reports of their own personality dysfunction would be informative to understanding if maternal and adolescent personality dysfunction demonstrate bidirectional associations and how they might relate to adolescent emotion regulation. Additional research could consider examining maternal LPFS reports in infancy and early childhood. Although these are populations that receive a lot of attention regarding how maternal factors influence the development of ES [49], no research to our knowledge has examined the LPFS specifically in this age group, which is important to consider alongside the commonly used trait-based measures [34].

## Conclusion

Despite the limitations, our study is an important contribution to the existing literature on the effects of maternal characteristics on adolescent emotion regulation. Specifically, we were able to demonstrate that maternal ES and interpersonal personality dysfunction are predictors of adolescent ES use, which has implications for preventing negative outcomes that may result from ES use in this age group. Although we must cautiously interpret these results due to our powering issue and more work is certainly needed, our study is an important first step in this area.

## Data Availability

Data that support the findings of this study are available from the corresponding author upon reasonable request.

## Abbreviations

ES: Emotion Suppression
CR: Cognitive Reappraisal
LPFS: Levels of Personality Functioning Scale
BPD: Borderline Personality Disorder
AMPD: Alternative Model of Personality Disorders
EM: Estimation Maximization
VIF: Variance Inflation Factor
CX: Condition Index
VP: Variance Proportions
